# Symptoms of Depression, Anxiety, and Stress Among Nursing Students and Coping Strategies Used

**DOI:** 10.1590/0034-7167-2025-0386

**Published:** 2026-08-21

**Authors:** Ana Paula Porto Cruz, Maria Clara Santana da Silva, Maria Rita Sousa da Silva, Bárbara Beatriz Coelho Lima, Maria Edileuza Soares Moura

**Affiliations:** I Universidade Federal de Rondonópolis. Rondonópolis, Mato Grosso, Brazil; II Universidade Estadual do Maranhão. Caxias, Maranhão, Brazil; III Universidade Federal do Piauí. Teresina, Piauí, Brazil; IV Centro Universitário Santo Agostinho. Teresina, Piauí, Brazil

**Keywords:** Adaptation, Psychological, Coping Skills, Students, Nursing, Mental Health, Psychic Symptoms., Adaptación Psicológica, Habilidades de Afrontamiento, Estudiantes de Enfermería, Salud Mental, Síntomas Psíquicos.

## Abstract

**Objectives:**

to analyze symptoms of depression, anxiety, and stress among nursing students and the coping strategies most frequently used.

**Methods:**

an observational, descriptive, and cross-sectional study with a quantitative approach, conducted with 81 students in the second semester of 2023. The Depression, Anxiety and Stress Scale-21 and the Coping Strategies Inventory were applied and analyzed using descriptive statistics for frequencies, Cronbach’s alpha and McDonald’s omega tests for instrument reliability, and Bartlett’s test of sphericity and Kaiser-Meyer-Olkin test for inventory adequacy.

**Results:**

symptoms of depression predominated at normal and mild levels, while anxiety and stress symptoms ranged from moderate to very severe. Emotionally focused coping strategies were used (distancing, escape and avoidance, and positive reappraisal).

**Conclusions:**

the frequency of symptoms of psychological distress and coping mechanisms used suggest the need for institutional actions that promote the development of psychosocial skills and emotional intelligence.

## RESUMEN

**Objetivos:**

analizar los síntomas de depresión, ansiedad y estrés en estudiantes de enfermería y las estrategias de afrontamiento más utilizadas.

**Métodos:**

estudio observacional, descriptivo y transversal con enfoque cuantitativo, realizado con 81 estudiantes durante el segundo semestre de 2023. Se aplicaron la Escala de Depresión, Ansiedad y Estrés-21 y el Inventario de Estrategias de Afrontamiento y se analizaron utilizando estadística descriptiva para frecuencias, pruebas alfa de Cronbach y omega de McDonald para confiabilidad del instrumento, y pruebas de esfericidad de Bartlett y Kaiser-Meyer-Olkin para adecuación del inventario.

**Resultados:**

los síntomas de depresión predominaron en niveles normales y leves, mientras que los síntomas de ansiedad y estrés variaron de moderados a muy graves. Se emplearon estrategias de afrontamiento centradas en las emociones (distanciamiento, escape y evitación, y reevaluación positiva).

**Conclusiones:**

la frecuencia de síntomas de distrés psicológico y los mecanismos de afrontamiento utilizados sugieren la necesidad de acciones institucionales que promuevan el desarrollo de habilidades psicosociales y la inteligencia emocional.

## INTRODUCTION

The entry of young adults into higher education is a complex process of adaptation to new academic, social, and emotional demands, directly impacting mental health. This entry is characterized by requirements related to autonomy in studies, time management, integration into new social groups, and formulation of professional expectations. In nursing courses, these demands tend to intensify due to the nature of the training, which is centered on care and responsibility for human life^(1)^.

According to the World Health Organization, mental health is defined as a state of well-being in which individuals are able to cope with everyday stressors, develop their potential, and contribute to society. This balance is influenced by biopsychosocial factors, and can be compromised by experiences of stigmatization, discrimination, and violations of rights^(2)^. In the context of nursing education, high competitiveness, assessment pressures, and contact with suffering and death can cause mental health disorders^(1)^.

Therefore, the application of coping strategies is fundamental for academic adaptation, as it allows for better management of adverse circumstances, more effective direction of efforts, and mitigation of internal and external stressors. When functional, these strategies can promote emotional regulation and long-term well-being. On the other hand, dysfunctional strategies, despite momentary relief, lead to cumulative detrimental effects over time^(3,4)^.

Therefore, when coping responses are ineffective, symptoms of depression, anxiety, and stress can emerge, compromising quality of life^(5)^. In this regard, the most frequent depressive symptoms in university students include impairments in reasoning ability, memorization, motivation, and interest in studies, as well as negative views and distortions of reality. These symptoms affect academic performance and encourage university dropout^(6,7)^.

From another perspective, the most prevalent manifestations associated with anxiety are restlessness, muscle stiffness, anticipation of activities, panic attacks, feelings of inadequacy, and demotivation. These symptoms represent psycho-emotional, neurocognitive, behavioral, and somatic barriers that compromise students’ physical and mental health^(8)^.

Additionally, stress symptoms manifest through psychophysiological indicators such as sleep disturbances, social isolation, impaired selective attention, anxiety, hypervigilance, recurring worries, and affective lability. When persistent, these can impair psychomotor coordination and overall cognitive performance^(9)^.

Although there have been advances in understanding the impacts of the university environment on mental health, gaps remain in literature regarding the integrated analysis of psychoemotional symptomatology and coping styles among nursing students. Existing studies are fragmented and insufficient to elucidate how coping mechanisms influence the modulation of symptoms of depression, anxiety, and stress, hindering the development of effective intervention strategies^(10,11)^.

Given the detrimental effects of symptoms of depression, anxiety, and stress in higher education, this study sought to analyze these symptoms and identify the coping strategies most used by nursing students at a public university in the state of Maranhão, Brazil. Thus, the investigation aligns with the third Sustainable Development Goal (SDG) of the United Nations 2030 Agenda, whose fourth target recognizes mental health as part of comprehensive health and intrinsic to sustainable human development. Therefore, understanding these aspects can contribute to the formulation of preventive and protective strategies in the university context that can extend to other areas and moments in these students’ lives^(12)^.

### Study relevance

This article is derived from the monograph entitled “Analysis of the level of depression, anxiety, stress among university students and coping strategies used”, presented to the State University of Maranhão for the Bachelor of Nursing course in 2024. The relevance of the study focuses on its contribution to the advancement of knowledge in nursing by highlighting aspects of the mental health of students and the coping strategies used, providing subsidies for the formulation of institutional policies to support undergraduates^(13)^.

## OBJECTIVES

To analyze symptoms of depression, anxiety, and stress among nursing students and the coping strategies most commonly used.

## METHODS

### Ethical aspects

This study was conducted in accordance with the ethical guidelines established by Resolution 510/2016 of the Brazilian National Health Council and approved by the university’s Research Ethics Committee. All participants electronically consented to their participation by signing the Informed Consent Form.

### Study design, period and location

This is an observational, descriptive, and cross-sectional study with a quantitative approach, developed based on STrengthening the Reporting of OBservational studies in Epidemiology recommendations, which guides the conduct and presentation of observational studies in epidemiology. The research was carried out from August to December 2023, with the target population being students regularly enrolled in the nursing course at a public university in the state of Maranhão, Brazil.

### Population and inclusion/exclusion criteria

The study population consisted of 151 students regularly enrolled in the nursing course. The sample included students aged 18 or older, enrolled between the first and tenth semesters of the course. Students with a prior diagnosis of psychiatric disorders, specifically anxiety and/or depression, were excluded.

For sample size calculation, an online calculator specifically for finite populations was used, considering a 95% confidence level and a 5% margin of error, resulting in an estimated sample of 109 participants. However, at the end of data collection, only 81 valid responses were obtained from the questionnaires.

### Study protocol

Data collection was carried out in the second half of 2023 using an electronic form created on Google Forms^®^. The link to access the questionnaire contained a presentation of the study, instructions for completion, and the Informed Consent Form (ICF), which had to be read and accepted by participants before beginning to complete the instruments. These instruments assessed sociodemographic variables, symptoms of depression, anxiety and stress, and coping strategies.

Considering the context of the suspension of in-person academic activities due to the faculty strike during the data collection period, the invitation to participate was conducted remotely and sent weekly for four consecutive weeks through institutional WhatsApp^®^ groups previously organized by students. This strategy aimed to ensure the reach of the target population and enable universal and autonomous access to the data collection instruments.

During the strike, in-person activities were completely interrupted. Although some professors maintained virtual classes, these did not exceed two weeks, a period during which all professors formally joined the strike. Consequently, no academic activities or assessments were carried out between August and December 2023, which may have contributed to the results obtained in this study.

To achieve the study objectives, a set of instruments with proven psychometric validity was used. Regarding participant sociodemographic characterization, a structured instrument, developed and validated by Mendonça (2020) based on investigations related to the thematic axis of this study, was applied, allowing for better contextualization of the sample^(14)^.

The Depression, Anxiety and Stress Scale-21 (DASS-21) was used to measure symptoms in the depression, anxiety, and stress dimensions. It is a self-report instrument with 21 questions, scored on a four-point Likert scale (0 - strongly disagree to 3 - strongly agree), developed by Lovibond and Lovibond (2004), and adapted to the Brazilian context by Vignola and Tucci (2014). Classification is based on the sum of the scores in each subscale: depression (normal, 0 to 9, and very severe, 28 or more); anxiety (normal, 0 to 7, and very severe, 20 or more); and stress (normal, 0 to 14, and very severe, 34 or more)^(15,16)^.

Additionally, to identify the most frequently used coping strategies, the Coping Strategies Inventory (CSI), developed by Lazarus and Folkman (1984) and adapted for Brazil by Savóia, Santana, and Mejias (1996), was applied. This instrument has 66 items, organized into eight dimensions or factors (coping, withdrawal, self-control, seeking social support, acceptance of responsibility, escape-avoidance, problem-solving, and positive reappraisal), scored using a four-point Likert scale (0 - not used, 3 - used extensively). The raw score is obtained by summing the items per factor, and the relative score, in percentage (0-100), describes the proportion of effort per strategy^(17,18)^. Its application contributed to the characterization of students’ coping profile and its relationship with symptoms evidenced by DASS-21.

### Analysis of results and statistics

The data collected via Google Forms^®^ were initially imported and organized in Microsoft Excel^®^, and subsequently exported to the Statistical Package for the Social Sciences^®^ version 25.0, where analyses were performed. Regarding students who were not enrolled in their regular academic year, i.e., those who declared they were taking courses from different semesters simultaneously, the classification criterion adopted for the analysis was the most advanced semester in which they were enrolled.

Descriptive statistical analysis, with calculation of absolute and relative frequencies, was employed for categorical variables. Means, standard deviations, measures of central tendency, and dispersion were used for continuous variables. To assess data collection instrument’s internal consistency, Cronbach’s alpha coefficient was applied, and Fisher’s exact test, appropriate for samples with low expected frequencies, was used for comparisons among groups.

An exploratory factor analysis of the CSI, composed of 66 items, was performed to assess its factorial structure in the study sample. The feasibility of this analysis was verified by Bartlett’s test of sphericity and the Kaiser-Meyer-Olkin index. The principal component extraction method was employed, and the component matrix was assessed to identify each item’s contribution. Thus, to simplify the data structure, remove redundancy, and reveal the underlying dimensions that explain most of the total variance of the data, 18 items were excluded due to criteria of low factor loading (no strong correlation in any of the main factors) and statistical inadequacy (significant loadings in several factors at the same time), and 48 items were considered valid and organized into eight main factors for the final analysis (latent dimensions that the analysis identified as the most important to explain the variance).

In addition to the tests described, in order to verify possible differences in the use of coping strategies among students in the initial years (1^st^ to 5^th^ semester) and final years of the course (6^th^ to 10^th^ semester), an independent t-test for unpaired samples was applied, considering the eight factorial variables previously identified in factor analysis. Additionally, McDonald’s omega coefficient was calculated to assess the reliability of the psychometric instruments. In all inferential statistical analyses, a significance level of 5% (p ≤ 0.05) was adopted.

## RESULTS

This study included 81 students, mostly aged between 18 and 35 years, with an average age of 19.4 years. There was a predominance of females (77.8%) and students enrolled in different periods of the nursing course. The majority identified as Catholic (55.6%), single (97.5%), and low-income (53.3%).

Concerning lifestyle habits, 46.9% reported occasionally dedicating time to leisure, while 53.1% considered their sleep quality unsatisfactory. Using social media for more than two hours a day was reported by 46.9% of participants, and 51.9% stated they engaged in physical activity more than three times a week. Furthermore, 91.4% did not have paid employment; 59.3% did not receive a scholarship; and 54.3% did not participate in extracurricular activities (Table 1).

**Table 1:** Sample sociodemographic characterization, Caxias, Maranhão, Brazil, 2025 (N=81)

Variables	n	%
**Sex**		
Female	63	77.8
Male	18	22.2
**Age group**		
18 - 19	21	26.0
20 - 24	49	60.5
25 - 30	10	12.3
30 - 35	01	1.2
**Course period**		
Initial years (1st - 5th)	47	58.0
Final years (6th - 10th)	34	42.0
**Marital status**		
Married	2	2.5
Single	79	97.5
**Social class**		
Lower	44	54.3
Middle	37	45.7
**Engages in paid activity**		
Yes	7	8.6
No	74	91.4
**Receives research grant**		
Yes	33	40.7
No	48	59.3
**Participates in extracurricular activities**		
Yes	37	45.7
No	44	54.3
**Sleep quality**		
Satisfactory	38	46.9
Unsatisfactory	43	53.1
**Dedication to leisure**		
Often	22	27.2
Occasionally	38	46.9
Rarely	21	25.9
**Physical exercise**		
Once a week	7	8.6
Twice a week	12	14.8
More than three times a week	42	51.9
I do not exercise	20	24.7
**Use of digital social network**		
More than five hours a day	35	43.2
More than two hours a day	38	46.9
Up to one hour a day	6	7.4
Never looks	2	2.5

Analysis of DASS-21 results showed that, among the three dimensions assessed, anxiety was the most frequent and severe symptom in the studied sample. While depression levels showed a slightly higher distribution in the normal/mild range (53.1%), stress levels were evenly distributed between normal/mild (49.4%) and moderate/very severe (50.6%). Approximately two-thirds of participants (67.9%) presented moderate to very severe levels of anxiety, with a notable prevalence of 34.6% at the very severe level, exceeding the very severe stress scores (7.4%) (Table 2).

**Table 2:** Scores of depression, anxiety, and stress levels among nursing students according to the Depression, Anxiety, and Stress Scale-21 and Cronbach’s alpha values for each subscale, Caxias, Maranhão, Brazil, 2025 (N=81)

Depression (Cronbach’s alpha 0.910)			Anxiety (Cronbach’s alpha 0.884)			Stress (Cronbach’s alpha 0.877)		
**Score**	**n**	**%**	**Score**	**n**	**%**	**Score**	**n**	**%**
Normal (0-9)	30	37.0	Normal (0-7)	20	24.7	Normal (0-14)	32	39.5
Mild (10-13)	13	16.1	Mild (8-9)	6	7.4	Mild (15-18)	8	9.9
Moderate (14-20)	13	16.1	Moderate (10-14)	15	18.5	Moderate (19-25)	16	19.8
Severe (21-27)	10	12.3	Severe (15-19)	12	14.8	Severe (26-33)	19	23.4
Very severe (28 or +)	15	18.5	Very severe (20 ou +)	28	34.6	Very severe (34 ou +)	6	7.4
Total	81	100	Total	81	100	Total	81	100

Comparative analysis of the DASS-21 among students in the initial and final years of the course did not reveal statistically significant differences in any of the subscales (p>0.05). The data, confirmed by Fisher’s exact test, indicate that the severity of emotional symptoms is homogeneous, regardless of the length of time spent in the course (Table 3).

**Table 3:** Level of depression, anxiety, and stress measured by the Depression, Anxiety, and Stress Scale-21 among nursing students in the early and later years of their course, using Fisher’s exact test for the subscale, Caxias, Maranhão, Brazil, 2025 (N=81)

Subscales	Initial years		Final years		*p* value
	n	%	n	%	
**Depression**					**0.067**
Normal (0-9)	16	34.0	14	41.1	
Mild (10-13)	6	12.8	7	20.6	
Moderate (14-20)	11	23.4	2	5.9	
Severe (21-27)	8	17.0	2	5.9	
Very severe (28 or +)	6	12.8	9	26.5	
Total	**47**	**100**	**34**	**100**	
**Anxiety**					**0.970**
Normal (0-7)	11	23.4	9	26.5	
Mild (8-9)	3	6.5	3	8.8	
Moderate (10-14)	9	19.1	6	17.6	
Severe (15-19)	8	17.0	4	11.8	
Very severe (20 or +)	16	34.0	12	35.3	
Total	**47**	**100**	**34**	**100**	
**Stress**					
Normal (0-14)	17	36.2	15	44.0	**0.234**
Mild (15-18)	4	8.5	4	11.8	
Moderate (19-25)	9	19.1	7	20.6	
Severe (26-33)	15	31.9	4	11.8	
Very severe (34 ou +)	2	4.3	4	11.8	
Total	**47**	**100**	**34**	**100**	

CSI descriptive analysis points to a preference for emotion-focused mechanisms over direct action. The most frequently used factor was escape-avoidance (=8.88; Med=9.00), followed by withdrawal (=8.28; Med=8.00). These scores reinforce students’ tendency to avoid direct coping with the stressor, a behavior further evidenced by the fact that the coping strategy had the lowest usage score (=4.63; Med=4.00). Even so, positive reappraisal (=7.77; Med=7.00) was the third highest score, indicating that although they avoid coping, participants seek to reinterpret stressful situations, attributing a new and positive meaning to them (Table 4).

**Table 4:** Eight coping factors and their respective items with mean and median scores for participants, Caxias, Maranhão, Brazil, 2025

Factors	Mean	Median
1 - Coping (06, 07, 17, 28, 34, 47)	4.63	4.00
2 - Withdrawal (12, 13, 15, 21, 41, 44, 62)	8.28	8.00
3 - Self-control (10, 14, 35, 43, 54)	6.30	6.00
4 - Social support (08, 18, 22, 31, 42, 45)	5.14	5.00
5 - Acceptance of responsibility (09, 25, 29, 51)	5.48	6.00
6 - Escape-avoidance (11, 16, 33, 40, 50, 58, 59)	8.88	9.00
7 - Problem-solving (01, 26, 39, 46, 48, 49, 52)	6.25	7.00
8 - Positive reappraisal (20, 23, 30, 36, 38, 56, 60)	7.77	7.00

The comparison of coping strategies between students in the initial and final years of the course did not show statistically significant differences in any of the eight factors. The means among groups, analyzed using an independent t-test, showed p-values greater than 0.05. Therefore, coping strategies were homogeneous throughout the course (Table 5).

**Table 5:** Independent t-test results for coping strategies, Caxias, Maranhão, Brazil, 2025

Factors	Initial years (n=47)		Final years (n=34)		*p* value
	Mean	Standard deviation	Mean	Standard deviation	
1 - Coping	4.70	3.482	4.53	3.744	0.832
2 - Withdrawal	8.68	5.022	7.74	4.158	0.372
3 - Self-control	6.79	3.759	5.62	2.719	0.108
4 - Social support	5.00	3.563	5.32	3.391	0.682
5 - Acceptance of responsibility	5.60	2.872	5.32	2.590	0.662
6 - Escape-avoidance	8.70	5.094	9.12	5.319	0.723
7 - Problem-solving	6.19	3.976	6.32	3.464	0.877
8 - Positive reappraisal	7.91	4.643	7.56	3.703	0.712

## DISCUSSION

The findings of this study revealed a predominance of mild depression symptoms and moderate/very severe anxiety and stress symptoms among participants. Simultaneously, the coping profile of these students was characterized by the use of emotion-focused coping strategies, in which withdrawal, escape-avoidance, and positive reappraisal were the most frequently used.

The symptom levels evidenced by the DASS-21 among participants in this study are similar to those of research conducted at another public institution, which revealed moderate levels of depressive symptoms, extremely severe anxiety, and severe stress. However, the aforementioned study was conducted during the SARS-CoV-2 pandemic, while the present survey took place in a post-pandemic context, but marked by a scenario of instability resulting from a professors’ strike, which may also have contributed to the results^(19)^.

Pressure to perform, coupled with self-criticism and insecurity, is linked to an increase in depressive symptoms among nursing students. Furthermore, moral distress caused by unmet personal or professional expectations can intensify this situation by generating negative self-directed emotions such as guilt and shame^(20,21)^.

In relation to anxiety symptoms, although they share risk factors with depressive symptoms, they present specific manifestations in the university context. The amount of content to be mastered in a short period of time and the difficulty in establishing boundaries between study and personal life favor states of mental hyperactivity, excessive worry, and a constant feeling of alertness^(22)^.

Furthermore, the stress levels of participants in this study were predominantly reported as moderate to severe. In contrast, a study conducted at a private university in southeastern Brazil showed low stress levels in its sample, which may be attributed to differences in the institutional setting, such as better academic infrastructure, greater availability of psycho-pedagogical support, and less exposure to situations of socioeconomic vulnerability^(23)^.

Academic stress can be exacerbated by initial factors, such as pressure to perform, which, combined with other factors like low frustration tolerance, triggers dysfunctional emotional responses. In demanding courses, such as nursing, continuous exposure to stressors compromises physical and mental recovery, favoring ruminative thoughts that mediate the relationship between chronic stress and depressive symptoms^(24,25)^.

Given the symptoms, CSI factor analysis did not reveal a significant difference in symptoms and strategies between the initial and final years of the course, indicating a homogeneous and continuous pattern of responses to stressors in this group. Such patterns may aggravate symptoms of depression, anxiety, and stress by favoring psychological distress and emotional overload maintenance. Although it denotes some level of resilience, the positive reappraisal, together with distancing strategies, demonstrates a compensatory attempt at the low use of more functional strategies, such as self-control, problem-solving, acceptance of responsibility, and seeking social support.

Coping strategies are classified into two functional categories: problem-focused and emotion-focused. Problem-focused strategies seek to modify or eliminate the source of stress, promoting effective long-term adaptation. In contrast, emotion-focused strategies aim to regulate the impacts of the stressful situation, providing immediate subjective relief, but with the potential for harm in chronic or recurring contexts^(3)^.

Among the emotion-centered strategies, the ones most used by participants in this study can be understood in light of the concept of aversive control, in which students tend to avoid or interrupt contact with aversive stimuli, whether external, such as poor academic performance, or internal, such as excessive self-criticism^(26)^. Thus, in the long term, such strategies are ineffective in reducing stress-related distress and may compromise self-efficacy, content assimilation, and academic performance. Meanwhile, problem-focused coping, such as positive reappraisal, promotes more functional adaptation^(27)^.

In the university context, mild stressors tend to be assessed as manageable, based on prior experiences or internalized cultural norms. More intense situations, such as the risk of failing a course, can generate automatic negative responses, requiring cognitive reappraisal for adaptive reinterpretation, which involves inhibiting dysfunctional thoughts and regulating emotional responses. Thus, positive reappraisal mitigates the impact of stress, promoting emotional balance^(28)^.

Most studies that assessed coping strategies among nursing undergraduate students were conducted during the pandemic or related to training programs, due to the uncertainties of those times. However, even in the face of such challenges, the coping strategies used were more effective than those in the present study^(29,30)^.

Thus, by interpreting the psychometric data in conjunction with the sociodemographic profile of the sample, a better understanding is obtained of the challenges faced by this group, composed predominantly of female students, with an average age of 19.4 years, single, without children, from low-income families, and living in shared households, consistent with the profile of students entering bachelor’s degree programs in nursing in Brazil^(19,31)^.

As for gender issues, it is important to remember the origins of nursing, deeply rooted in ethical and humanitarian principles. However, due to its historical development within a context that linked the act of caring to women and charity, this paradigm resulted in its sociocultural stigmatization^(32)^. This distorted social perception contributes to devaluating the course choice, sometimes considered less prestigious, at odds with the real complexity and relevance of its contributions to individuals’ overall health^(33)^.

In addition to the issues mentioned, students in this field face structural obstacles, especially related to curriculum organization and socioeconomic realities. The curriculum (full-time in the location of this study) and the difficulty of reconciling studies with paid work, coupled with a scarcity of financial aid, impose additional barriers, particularly for students in situations of socioeconomic vulnerability, who depend almost exclusively on support networks to ensure their continued enrollment in the course^(34)^.

In addition to the mandatory curriculum, involvement in extracurricular activities (with little participation in this study), despite being an additional requirement, contributes to strengthening the sense of belonging and identification with the profession, by fostering the development of self-confidence in the fundamental technical and theoretical skills necessary for entering the job market^(35)^.

Regarding protective factors, students highlighted a good routine of physical exercise and dedication to leisure, but high levels of stress were still observed. These results contrast with evidence in the literature, which indicates that these practices alleviate psychological distress. However, variables such as the type of activity performed, the amount of time dedicated, and the form of engagement can influence relaxation perception^(36-38)^.

In addition to this scenario, participants in this study considered sleep quality unsatisfactory. In this regard, scientific evidence indicates that unsatisfactory sleep can significantly compromise academic performance as well as have adverse effects on mental health. These impacts include elevated stress levels, impaired concentration, and reduced optimism, which influence performance and perception of the chosen course of study^(39,40)^.

It is also understood that sleep quality is negatively impacted by continuous exposure to digital stimuli resulting from the massive use of screens for academic and social purposes, which predisposes individuals to dysfunctional stress responses and compromises their emotional state^(41)^. Furthermore, social media, in particular, contributes to an increase in anxiety symptoms, as it functions as a form of escapism. Upon returning to reality, a person feels unproductive, has inefficient time management, and is distressed^(42)^.

Although the literature does not establish a specific age for the analysis and modulation of coping strategies, it is suggested that early exposure to the subject may contribute to the consolidation of more adaptive behavioral patterns for self-knowledge and personal development^(3)^.

In light of this analysis and discussion, the importance of public higher education institutions, more specifically Departments of Nursing, adopting a comprehensive student healthcare plan is reinforced, with a view to expanding the provision of psycho-pedagogical and psychological support aimed at promoting more effective coping strategies and strengthening socio-emotional skills.

Additionally, it is crucial to empower professors to recognize and address vulnerabilities among students, ensuring their early detection and effective referral to support services, as well as the inclusion of activities aimed at promoting well-being in the curriculum. Thus, by fostering the development of internal coping resources and structural support, these initiatives can contribute to training more autonomous and mentally healthy professionals, in line with the fourth target of the third SDG^(10)^.

### Study limitations

This study presents some limitations that should be considered when interpreting the results. The sample consisted of a small number of students from a single public institution, which may restrict the generalization of the findings to other academic settings. The cross-sectional design only allows for the identification of associations and does not establish causal relationships among the variables assessed. Data collection, through self-reported questionnaires, may also be subject to biases, such as in the interpretation of the questionnaires. Furthermore, the specific post-pandemic context, associated with faculty strike, may have influenced the levels of symptoms of depression, anxiety, and stress, making it difficult to separate the effects of these conditions from usual factors in the academic environment.

For future research, it is suggested that longitudinal studies be conducted to assess the effectiveness of interventions to promote functional coping, monitoring their impact on symptoms of depression, anxiety, and stress in a larger sample.

### Contributions to health, nursing, or public policy

By identifying significant levels of depressive, anxious, and stress symptoms, and the use of emotionally focused coping strategies among nursing students at a university in the state of Maranhão, Brazil, coupled with the analysis of sociodemographic data, this study provides important insights for the development of institutional policies that promote comprehensive student health support, with a focus on mental health. Thus, this situational diagnosis can encourage the search for intervention strategies aimed at mitigating psychological distress among students and indirectly contribute to the improvement of nursing training programs, which should prioritize the overall well-being of future professionals so that they can face adversities during their university experiences with greater autonomy and be better adapted to the demands of their professional activity.

## CONCLUSIONS

This study demonstrated the significant presence of symptoms of depression, anxiety, and stress among nursing students, while highlighting the predominance of dysfunctional coping strategies. Therefore, it emphasizes the need for institutional actions that promote the development of psychosocial skills and emotional intelligence, through the implementation of effective, continuous, and accessible psycho-pedagogical and psychological support programs, as well as activities that value professional identity and promote well-being, aiming at training nursing professionals with greater psycho-emotional mastery and the achievement of the fourth goal of the third SDG.

## Data Availability

The research data are available within the article.
