# Supplementary material for: Symptoms of Depression, Anxiety, and Stress Among Nursing Students and Coping Strategies Used
Source: Rev Bras Enferm. 2026 Aug 21;79:e20250386. doi: 10.1590/0034-7167-2025-0386 (PMC13497355; doi:10.1590/0034-7167-2025-0386)
Supplement: Translation

# Translation: Sintomas de Depressão, Ansiedade, Estresse entre Estudantes de Enfermagem e Estratégias de *Coping* Utilizadas

**DOI:** 10.1590/0034-7167-2025-0386pt

**Authors:** Ana Paula Porto Cruz, Maria Clara Santana da Silva, Maria Rita Sousa da Silva, Bárbara Beatriz Coelho Lima, Maria Edileuza Soares Moura

**Affiliations:** I Universidade Federal de Rondonópolis. Rondonópolis, Mato Grosso, Brasil; II Universidade Estadual do Maranhão. Caxias, Maranhão, Brasil; III Universidade Federal do Piauí. Teresina, Piauí, Brasil; IV Centro Universitário Santo Agostinho. Teresina, Piauí, Brasil

**Keywords:** Adaptação Psicológica, Estratégias de Enfrentamento, Estudantes de Enfermagem, Saúde Mental, Sintomas Psíquicos.

## Abstract

**Objetivos:**

analisar sintomas de depressão, ansiedade e estresse entre estudantes de enfermagem e as estratégias de *coping* mais utilizadas.

**Métodos:**

estudo observacional, descritivo e transversal, com abordagem quantitativa, conduzido com 81 estudantes no segundo semestre de 2023. Aplicaram-se a Escala de Depressão, Ansiedade e Estresse-21 e o Inventário de Estratégias de *Coping*, analisados por estatística descritiva, para frequências, testes alfa de Cronbach e ômega de McDonald, para confiabilidade dos instrumentos, e testes de Esfericidade de Bartlett e Kaiser-Meyer-Olkin, para adequação do inventário.

**Resultados:**

predominaram sintomas de depressão em níveis normais e leves, e de ansiedade e estresse em níveis moderados a muito graves. Utilizaram-se estratégias de *coping* com foco emocional (afastamento, fuga e esquiva, e reavaliação positiva).

**Conclusões:**

a frequência de sintomas de sofrimento psíquico e as formas de enfrentamento utilizadas sugerem a necessidade de ações institucionais que promovam o desenvolvimento de habilidades psicossociais e de inteligência emocional.

## INTRODUÇÃO

A inserção de adultos jovens no ensino superior configura-se como um processo complexo de adaptação a novas demandas acadêmicas, sociais e emocionais, impactando diretamente a saúde mental. Esta inserção é caracterizada por exigências relacionadas à autonomia nos estudos, gestão do tempo, integração em novos grupos sociais e formulação de expectativas profissionais. No curso de enfermagem, tais demandas tendem a se intensificar devido à natureza da formação, centrada no cuidado e na responsabilidade com a vida humana^(1)^.

De acordo com a Organização Mundial da Saúde, saúde mental é definida como um estado de bem-estar no qual o indivíduo consegue lidar com os estressores cotidianos, desenvolver seu potencial e contribuir para a sociedade. Esse equilíbrio é influenciado por fatores biopsicossociais, e pode ser comprometido por experiências de estigmatização, discriminação e violações de direitos^(2)^. No âmbito da formação em enfermagem, a alta competitividade, pressões avaliativas e o contato com o sofrimento e o morrer podem causar desordens à saúde mental^(1)^.

Assim, a aplicação de estratégias de *coping* ou enfrentamento é fundamental para a adaptação acadêmica, uma vez que possibilita administrar melhor circunstâncias adversas, direcionar esforços de forma mais eficaz e mitigar demandas estressoras internas e externas. Quando funcionais, essas estratégias podem promover a regulação emocional e bem-estar em longo prazo. Por outro lado, estratégias disfuncionais, apesar do alívio momentâneo, acarretam efeitos prejudiciais cumulativos ao longo do tempo^(3,4)^.

Portanto, quando as respostas de enfrentamento não são eficazes, sintomas de depressão, ansiedade e estresse podem emergir, comprometendo a qualidade de vida^(5)^. Nesse sentido, os sintomas depressivos mais frequentes em universitários incluem prejuízos na capacidade de raciocínio, memorização, motivação e interesse pelos estudos, além de visão negativa e distorções da realidade. Tais sintomas afetam o desempenho acadêmico e incitam a evasão universitária^(6,7)^.

Sob outro enfoque, as manifestações associadas à ansiedade mais predominantes são inquietação, rigidez muscular, antecipação de atividades, crises de pânico, sensação de incapacidade e desmotivação. Esses quadros representam barreiras de ordem psicoemocional, neurocognitiva, comportamental e somática que comprometem a saúde física e mental dos estudantes^(8)^.

Adicionalmente, os sintomas de estresse se manifestam por meio de indicadores psicofisiológicos, como distúrbios do sono, isolamento social, comprometimento da atenção seletiva, angústia, hipervigilância, preocupações recorrentes e labilidade afetiva. Quando persistentes, podem prejudicar a coordenação psicomotora e o desempenho cognitivo global^(9)^.

Embora existam avanços na compreensão dos impactos do ambiente universitário na saúde mental, persistem lacunas na literatura quanto à análise integrada da sintomatologia psicoemocional e dos estilos de *coping* entre estudantes de enfermagem. Estudos existentes são fragmentados e insuficientes para elucidar como mecanismos de enfrentamento influenciam a modulação dos sintomas de depressão, ansiedade e estresse, dificultando a elaboração de estratégias interventivas eficazes^(10,11)^.

Diante dos efeitos deletérios dos sintomas de depressão, ansiedade e estresse no ensino superior, este estudo buscou analisar esses sintomas e identificar as estratégias de *coping* mais utilizadas por discentes de enfermagem de uma universidade pública do estado do Maranhão, Brasil. Assim, a investigação alinha-se ao terceiro Objetivo de Desenvolvimento Sustentável (ODS) da Agenda 2030 da Organização das Nações Unidas, cuja quarta meta reconhece a saúde mental como parte da saúde integral e intrínseca ao desenvolvimento humano sustentável. Portanto, compreender tais aspectos pode contribuir com a formulação de estratégias preventivas e protetivas no contexto universitário que podem se estender às demais áreas e momentos da vida desses estudantes^(12)^.

### Relevância do estudo

O presente artigo é derivado da Monografia intitulada “Análise do nível de Depressão, Ansiedade, Estresse entre Universitários e Estratégias de Coping Utilizadas”, apresentada à Universidade Estadual do Maranhão no curso de Bacharelado em Enfermagem em 2024. A relevância do estudo concentra-se na contribuição para o avanço do conhecimento em Enfermagem ao evidenciar aspectos da saúde mental de estudantes e as estratégias de enfrentamento utilizadas, fornecendo subsídios para a formulação de políticas institucionais de apoio ao graduando^(13)^.

## OBJETIVOS

Analisar sintomas de depressão, ansiedade e estresse entre estudantes de enfermagem e as estratégias de *coping* mais utilizadas.

## MÉTODOS

### Aspectos éticos

Este estudo foi conduzido em conformidade com as diretrizes éticas estabelecidas pela Resolução nº 510/2016 do Conselho Nacional de Saúde e aprovado pelo Comitê de Ética em Pesquisa da universidade. Todos os participantes consentiram eletronicamente sua participação por meio da assinatura do Termo de Consentimento Livre e Esclarecido (TCLE).

### Desenho, período e local do estudo

Trata-se de estudo observacional, do tipo descritivo e transversal, com abordagem quantitativa, desenvolvido com base nas recomendações da diretriz *STrengthening the Reporting of OBservational studies in Epidemiology*, que orienta a condução e apresentação de estudos observacionais em epidemiologia. A pesquisa foi realizada no período de agosto a dezembro de 2023, tendo como população-alvo os estudantes regularmente matriculados no curso de enfermagem de uma universidade pública do estado do Maranhão, Brasil.

### População e critérios de inclusão e exclusão

A população do estudo foi composta por 151 estudantes regularmente matriculados no curso de enfermagem. Para compor a amostra, foram incluídos estudantes com idade igual ou superior a 18 anos cursando entre o primeiro e o décimo período do curso. Foram excluídos aqueles que apresentavam diagnóstico prévio de transtornos psiquiátricos, especificamente ansiedade e/ou depressão.

Para o dimensionamento amostral, foi utilizada uma calculadora *online* específica para populações finitas, considerando um nível de confiança de 95% e margem de erro de 5%, o que resultou em uma amostra estimada de 109 participantes. No entanto, ao final da coleta de dados, foram obtidas 81 respostas válidas aos questionários aplicados.

### Protocolo do estudo

A coleta de dados foi realizada no segundo semestre de 2023, através de um formulário eletrônico elaborado na plataforma *Google Forms^®^*. O *link* de acesso ao questionário continha a apresentação do estudo, orientações para preenchimento e o TCLE, o qual deveria ser lido e aceito pelo participante antes do início do preenchimento dos instrumentos, nos quais foram avaliadas as variáveis sociodemográficas, sintomas de depressão, ansiedade e estresse, e estratégias de *coping*.

Considerando o contexto de paralisação das atividades acadêmicas presenciais devido à greve docente durante o período de coleta, o convite à participação foi realizado de forma remota e enviado semanalmente, durante quatro semanas consecutivas, por meio de grupos institucionais de *WhatsApp^®^* previamente organizados pelos estudantes. Essa estratégia visou garantir o alcance da população-alvo e possibilitar o acesso universal e autônomo aos instrumentos de coleta.

Durante a paralisação, as atividades presenciais foram completamente interrompidas. Embora alguns professores tenham mantido aulas virtuais, estas não ultrapassaram duas semanas, período em que todos os docentes aderiram formalmente à greve. Consequentemente, não foram realizadas atividades acadêmicas nem avaliações entre agosto e dezembro de 2023, o que pode ter contribuído com os resultados obtidos neste estudo.

Para alcançar os objetivos do estudo, utilizou-se um conjunto de instrumentos de validade psicométrica comprovada. No que tange à caracterização sociodemográfica dos participantes, foi aplicado um instrumento estruturado, desenvolvido e validado por Mendonça (2020) com base em investigações relacionadas ao eixo temático deste estudo, permitindo melhor contextualização da amostra^(14)^.

A Escala de Depressão, Ansiedade e Estresse-21 (DASS-21) foi utilizada para mensurar os sintomas nas dimensões depressão, ansiedade e estresse. Trata-se de instrumento de autorrelato com 21 questões, pontuado em uma escala do tipo Likert de quatro pontos (0 - discordo totalmente a 3 - concordo totalmente), desenvolvido por Lovibond e Lovibond (2004), e adaptado ao contexto brasileiro por Vignola e Tucci (2014). A classificação é feita de acordo com a soma da pontuação em cada subescala: depressão (normal, de 0 a 9, e muito grave, de 28 ou mais); ansiedade (normal, de 0 a 7, e muito grave, de 20 ou mais); e estresse (normal, de 0 a 14, e muito grave, de 34 ou mais)^(15,16)^.

Adicionalmente, para identificar as estratégias de *coping* mais utilizadas, aplicou-se o Inventário de Estratégias de *Coping* (IEC), desenvolvido por Lazarus e Folkman (1984) e adaptado para o Brasil por Savóia, Santana e Mejias (1996). Trata-se de instrumento com 66 itens, organizados em oito dimensões ou fatores (confronto, afastamento, autocontrole, busca por suporte social, aceitação de responsabilidade, fuga e esquiva, resolução de problemas, e reavaliação positiva), pontuados por uma escala Likert de quatro pontos (0 - não usei a 3 - usei em grande quantidade). O escore bruto é obtido pela soma dos itens por fator, e o escore relativo, em porcentagem (0-100), descreve a proporção de esforços por estratégia^(17,18)^. Sua aplicação contribuiu para a caracterização do perfil de *coping* dos discentes e sua relação com os sintomas evidenciados pela DASS-21.

### Análise dos resultados e estatísticas

Os dados coletados via plataforma *Google Forms^®^* foram inicialmente importados e organizados no *software Microsoft Excel* ^®^, sendo posteriormente exportados para o programa *Statistical Package for the Social Sciences* ^®^, versão 25.0, no qual foram realizadas as análises. Quanto aos discentes desperiodizados, ou seja, que declararam estar cursando disciplinas de diferentes períodos simultaneamente, o critério de classificação adotado para a análise foi o período mais avançado em que estavam matriculados.

Empregou-se análise estatística descritiva, com cálculo de frequências absolutas e relativas, para as variáveis categóricas. Utilizaram-se médias, desvios padrão, medidas de tendência central e dispersão para variáveis contínuas. Com o objetivo de avaliar a consistência interna do instrumento de coleta, foi aplicado o coeficiente alfa de Cronbach, e para comparação entre grupos, empregou-se o teste exato de Fisher, apropriado para amostras com frequências esperadas reduzidas.

A análise fatorial exploratória do IEC, composto por 66 itens, foi realizada para avaliar sua estrutura fatorial na amostra do estudo. A viabilidade desta análise foi verificada pelos testes de esfericidade de Bartlett e pelo índice Kaiser-Meyer-Olkin. O método de extração por componentes principais foi empregado, e a matriz de componentes foi avaliada para identificar a contribuição de cada item. Assim, para simplificar a estrutura dos dados, remover redundância e revelar as dimensões subjacentes que explicam a maior parte da variância total dos dados, 18 itens foram excluídos devido a critérios de baixa carga fatorial (sem correlação forte em nenhum dos fatores principais) e inadequação estatística (cargas significativas em vários fatores ao mesmo tempo), e 48 itens foram considerados válidos e organizados em oito fatores principais para a análise final (dimensões latentes que a análise identificou como as mais importantes para explicar a variância).

Além dos testes descritos, a fim de verificar possíveis diferenças no uso das estratégias de *coping* entre discentes dos anos iniciais (1º ao 5º período) e finais do curso (6º ao 10º período), foi aplicado o teste t independente para amostras não pareadas, considerando as oito variáveis fatoriais previamente identificadas na análise fatorial. Adicionalmente, foi calculado o coeficiente ômega de McDonald para avaliar a fidedignidade dos instrumentos psicométricos. Em todas as análises estatísticas inferenciais, foi adotado um nível de significância de 5% (p ≤ 0,05).

## RESULTADOS

Este estudo contou com a participação de 81 estudantes, a maioria com idade entre 18 e 35 anos, e média de 19,4 anos. Observou-se predominância do sexo feminino (77,8%) e de estudantes matriculados em diferentes períodos do curso de enfermagem. A maioria se identificou como católica (55,6%), solteira (97,5%) e de baixa renda (53,3%).

Quanto aos hábitos de vida, 46,9% relataram dedicar tempo ao lazer ocasionalmente, enquanto 53,1% consideraram sua qualidade do sono insatisfatória. O uso de redes sociais por mais de duas horas diárias foi relatado por 46,9% dos participantes, e 51,9% afirmaram praticar atividade física mais de três vezes por semana. Além disso, 91,4% não exerciam atividade remunerada; 59,3% não recebiam bolsa de estudo; e 54,3% não participavam de atividades extracurriculares (Tabela 1).

**Tabela 1:** Caracterização sociodemográfica da amostra, Caxias, Maranhão, Brasil, 2025 (N=81)

Variáveis	n	%
**Sexo**		
Feminino	63	77,8
Masculino	18	22,2
**Faixa etária**		
18 - 19	21	26,0
20 - 24	49	60,5
25 - 30	10	12,3
30 - 35	01	1,2
**Período no curso**		
Anos iniciais (1° - 5°)	47	58,0
Anos finais (6° - 10°)	34	42,0
**Estado civil**		
Casado (a)	2	2,5
Solteiro (a)	79	97,5
**Classe social**		
Baixa	44	54,3
Média	37	45,7
**Desenvolve atividade remunerada**		
Sim	7	8,6
Não	74	91,4
**Recebe bolsa de pesquisa**		
Sim	33	40,7
Não	48	59,3
**Participa de atividade extra curricular**		
Sim	37	45,7
Não	44	54,3
**Qualidade do sono**		
Satisfatório	38	46,9
Insatisfatório	43	53,1
**Dedicação ao lazer**		
Frequentemente	22	27,2
Ocasionalmente	38	46,9
Raramente	21	25,9
**Prática de exercício físico**		
Uma vez por semana	7	8,6
Duas vezes por semana	12	14,8
Mais de três vezes por semana	42	51,9
Não pratico exercício físico	20	24,7
**Uso de rede social digital**		
Mais de cinco horas por dia	35	43,2
Mais de duas horas por dia	38	46,9
Até uma hora por dia	6	7,4
Nunca olha	2	2,5

A análise dos resultados da DASS-21 demonstrou que, entre as três dimensões avaliadas, a ansiedade foi o sintoma mais frequente e com maior gravidade na amostra estudada. Enquanto o nível de depressão apresentou uma distribuição ligeiramente maior nas faixas normal/leve (53,1%), o nível de estresse se distribuiu de forma equilibrada entre normal/leve (49,4%) e moderado/muito grave (50,6%). Cerca de dois terços dos participantes (67,9%) apresentaram níveis moderado a muito grave de ansiedade, com uma prevalência notável de 34,6% no nível muito grave, superando os escores de estresse muito grave (7,4%) (Tabela 2).

**Tabela 2:** Escores dos níveis de depressão, ansiedade e estresse dos estudantes de enfermagem segundo a Escala de Depressão, Ansiedade e Estresse-21 e valores do alfa de Cronbach de cada subescala, Caxias, Maranhão, Brasil, 2025 (N=81)

Depressão (Alfa de Cronbach 0,910)			Ansiedade (Alfa de Cronbach 0,884)			Estresse (Alfa de Cronbach 0,877)		
**Escore**	**n**	**%**	**Escore**	**n**	**%**	**Escore**	**n**	**%**
Normal (0-9)	30	37,0	Normal (0-7)	20	24,7	Normal (0-14)	32	39,5
Leve (10-13)	13	16,1	Leve (8-9)	6	7,4	Leve (15-18)	8	9,9
Moderada (14-20)	13	16,1	Moderada (10-14)	15	18,5	Moderada (19-25)	16	19,8
Grave (21-27)	10	12,3	Grave (15-19)	12	14,8	Grave (26-33)	19	23,4
Muito grave (28 ou +)	15	18,5	Muito grave (20 ou +)	28	34,6	Muito grave (34 ou +)	6	7,4
Total	81	100	Total	81	100	Total	81	100

A análise comparativa da DASS-21 entre estudantes dos anos iniciais e finais do curso não revelou diferenças estatisticamente significativas em nenhuma das subescalas (p>0,05). Os dados, confirmados pelo teste exato de Fisher, indicam que a gravidade dos sintomas emocionais é homogênea, independentemente do tempo de permanência no curso (Tabela 3).

**Tabela 3:** Nível de depressão, ansiedade e estresse pela Escala de Depressão, Ansiedade e Estresse-21 dos estudantes de enfermagem nos anos iniciais e finais do curso utilizando teste exato de Fisher da subescala, Caxias, Maranhão, Brasil, 2025 (N=81)

Subescalas	Anos iniciais		Anos finais		Valor de *p*
	n	%	n	%	
**Depressão**					**0,067**
Normal (0-9)	16	34,0	14	41,1	
Leve (10-13)	6	12,8	7	20,6	
Moderada (14-20)	11	23,4	2	5,9	
Grave (21-27)	8	17,0	2	5,9	
Muito grave (28 ou +)	6	12,8	9	26,5	
Total	**47**	**100**	**34**	**100**	
**Ansiedade**					**0,970**
Normal (0-7)	11	23,4	9	26,5	
Leve (8-9)	3	6,5	3	8,8	
Moderada (10-14)	9	19,1	6	17,6	
Grave (15-19)	8	17,0	4	11,8	
Muito grave (20 ou +)	16	34,0	12	35,3	
Total	**47**	**100**	**34**	**100**	
**Estresse**					
Normal (0-14)	17	36,2	15	44,0	**0,234**
Leve (15-18)	4	8,5	4	11,8	
Moderada (19-25)	9	19,1	7	20,6	
Grave (26-33)	15	31,9	4	11,8	
Muito grave (34 ou +)	2	4,3	4	11,8	
Total	**47**	**100**	**34**	**100**	

A análise descritiva do IEC aponta para uma predileção por mecanismos focados na emoção em detrimento da ação direta. O fator mais utilizado foi fuga e esquiva (=8,88; Med=9,00), seguido por afastamento (=8,28; Med=8,00). Tais escores reforçam a tendência dos estudantes em evitar o confronto direto com o estressor, um comportamento ainda mais evidente pelo fato de a estratégia confronto ter apresentado o menor escore de utilização (=4,63; Med=4,00). Ainda assim, a reavaliação positiva (=7,77; Med= 7,00) foi o terceiro maior escore, o que indica que embora evitem o confronto, os participantes buscam reinterpretar as situações estressantes, atribuindo-lhes um significado novo e positivo (Tabela 4).

**Tabela 4:** Oito fatores do *coping* e seus respectivos itens com média e mediana dos participantes, Caxias, Maranhão, Brasil, 2025

Fatores	Média	Mediana
1 - Confronto (06, 07, 17, 28, 34, 47)	4,63	4,00
2 - Afastamento (12, 13, 15, 21, 41, 44, 62)	8,28	8,00
3 - Autocontrole (10, 14, 35, 43, 54)	6,30	6,00
4 - Suporte social (08, 18, 22, 31, 42, 45)	5,14	5,00
5 - Aceitação de responsabilidade (09, 25, 29, 51)	5,48	6,00
6 - Fuga e esquiva (11, 16, 33, 40, 50, 58, 59)	8,88	9,00
7 - Resolução de problemas (01, 26, 39, 46, 48, 49, 52)	6,25	7,00
8 - Reavaliação positiva (20, 23, 30, 36, 38, 56, 60)	7,77	7,00

A comparação das estratégias de *coping* entre os estudantes dos anos iniciais e finais do curso não evidenciou diferenças estatisticamente significativas em nenhum dos oito fatores. As médias entre os grupos, analisadas por meio do teste t independente, apresentaram valores de *p* superiores a 0,05. Portanto, as estratégias de enfrentamento foram homogêneas ao longo do curso (Tabela 5).

**Tabela 5:** Resultados do teste t independente para as estratégias de *coping,* Caxias, Maranhão, Brasil, 2025

Fatores	Anos iniciais (n=47)		Anos finais (n=34)		Valor de *p*
	Média	Desvio padrão	Média	Desvio padrão	
1 - Confronto	4,70	3,482	4,53	3,744	0,832
2 - Afastamento	8,68	5,022	7,74	4,158	0,372
3 - Autocontrole	6,79	3,759	5,62	2,719	0,108
4 - Suporte social	5,00	3,563	5,32	3,391	0,682
5 - Aceitação de responsabilidade	5,60	2,872	5,32	2,590	0,662
6 - Fuga e esquiva	8,70	5,094	9,12	5,319	0,723
7 - Resolução de problemas	6,19	3,976	6,32	3,464	0,877
8 - Reavaliação positiva	7,91	4,643	7,56	3,703	0,712

## DISCUSSÃO

Os achados do presente estudo revelaram a predominância de sintomas de depressão, em nível normal/leve, e de ansiedade e estresse, em nível moderado/muito grave, entre os participantes. Paralelamente, o perfil de enfrentamento desses estudantes foi caracterizado pelo uso de estratégias de *coping* com foco na emoção, no qual o afastamento, a fuga e esquiva e a reavaliação positiva foram as mais utilizadas.

Os níveis dos sintomas evidenciados pela DASS-21 entre os participantes deste estudo são próximos aos de pesquisa conduzida em outra instituição pública que revelou níveis moderados de sintomas depressivos, extremamente severos de ansiedade e severos de estresse. Contudo, o estudo supramencionado foi realizado durante o período da pandemia de SaRS-CoV-2, enquanto o presente levantamento ocorreu em um contexto pós-pandêmico, mas marcado por um cenário de instabilidade decorrente de uma greve docente, o que também pode ter contribuído com os resultados^(19)^.

A pressão por desempenho, associada à autocrítica e insegurança, está relacionada ao aumento de sintomas depressivos entre estudantes de enfermagem. Além disso, o sofrimento moral ocasionado por expectativas pessoais ou profissionais não atendidas pode intensificar esse quadro, ao gerar emoções autodirigidas negativas como culpa e vergonha^(20,21)^.

Quanto aos sintomas ansiosos, embora compartilhem fatores de risco com os depressivos, apresentam manifestações específicas no contexto universitário. A quantidade de conteúdos a serem dominados em curto intervalo de tempo e a dificuldade em estabelecer limites entre estudo e vida pessoal favorecem estados de hiperatividade mental, preocupação excessiva e constante sensação de alerta^(22)^.

Outrossim, os níveis de estresse dos participantes deste estudo foram apontados predominantemente como moderado e grave. Em contrassenso, estudo realizado em universidade particular na região Sudeste do Brasil retratou baixos níveis de estresse em sua amostra, o que pode ser atribuído a diferenças no cenário institucional, como melhor infraestrutura acadêmica, maior oferta de suporte psicopedagógico e menor exposição a situações de vulnerabilidade socioeconômica^(23)^.

O estresse acadêmico pode ser agravado por fatores iniciais, como a pressão por desempenho, que, acumulada a outros fatores, como a baixa tolerância à frustração, desencadeia respostas emocionais disfuncionais. Em cursos de alta demanda, como enfermagem, a exposição contínua a estressores compromete a recuperação física e mental, favorecendo pensamentos ruminativos, que mediam a relação entre estresse crônico e sintomas depressivos^(24,25)^.

Diante dos sintomas, a análise fatorial do IEC não revelou diferença significativa nos sintomas e estratégias entre os anos iniciais e finais do curso, apontando um padrão de respostas aos estressores homogêneo e contínuo neste grupo. Tais padrões podem agravar os sintomas de depressão, ansiedade e estresse por favorecem a manutenção do sofrimento psíquico e a sobrecarga emocional. Embora denote algum nível de resiliência, a reavaliação positiva em conjunto com as estratégias de distanciamento demonstra uma tentativa compensatória à baixa utilização de estratégias mais funcionais, como o autocontrole, resolução de problemas, aceitação de responsabilidade e busca por apoio social.

As estratégias de *coping* são classificadas em duas categorias funcionais: centradas no problema; e centradas na emoção. Estratégias focadas no problema buscam modificar ou eliminar a fonte do estresse, favorecendo a adaptação eficaz em longo prazo. Em contrapartida, estratégias centradas na emoção visam regular impactos decorrentes da situação estressora, proporcionando alívio subjetivo imediato, mas com potencial de prejuízo em contextos crônicos ou recorrentes^(3)^.

Entre as estratégias centradas na emoção, as mais utilizadas pelos participantes deste estudo podem ser compreendidas à luz do conceito de controle aversivo, em que o estudante tende a evitar ou interromper o contato com estímulos aversivos, sejam externos, como o baixo desempenho acadêmico, ou internos, como a autocrítica excessiva^(26)^. Assim, a longo prazo, tais estratégias são ineficazes na redução da angústia relacionada ao estresse, e podem comprometer a percepção de autoeficácia, assimilação de conteúdos e desempenho acadêmico. Enquanto isso, o enfrentamento com foco no problema, como a reavaliação positiva, promove adaptação mais funcional^(27)^.

No contexto universitário, estressores leves tendem a ser avaliados como gerenciáveis, com base em experiências prévias ou normas culturais internalizadas. Já situações mais intensas, como o risco de reprovação, podem gerar respostas negativas automáticas, exigindo reavaliação cognitiva para que sejam reinterpretadas de forma adaptativa, o que envolve a inibição de pensamentos disfuncionais e a regulação da resposta emocional. Assim, a reavaliação positiva atenua o impacto do estresse, favorecendo o equilíbrio emocional^(28)^.

A maioria dos estudos que avaliaram estratégias de enfrentamento por graduandos de enfermagem foram realizados durante o período pandêmico ou relacionados a treinamentos, devido às incertezas destes momentos. No entanto, ainda que diante de tais desafios, as estratégias de *coping* utilizadas foram mais funcionais que as do presente estudo^(29,30)^.

Assim, ao interpretar os dados psicométricos em conjunto com o perfil sociodemográfico da amostra, obtém-se uma melhor compreensão dos desafios enfrentados por este grupo, composto predominantemente por estudantes do sexo feminino, com idade média de 19,4 anos, solteiros, sem filhos, de baixa renda e em coabitação familiar, compatível com o perfil de ingressantes nos cursos de bacharelado em enfermagem no Brasil^(19,31)^.

Quanto às questões de gênero, é importante rememorar a origem da enfermagem, profundamente enraizada em princípios éticos e humanitários. Mas devido ao desenvolvimento histórico em um contexto que atrelava o ato de cuidar às mulheres e à caridade, tal paradigma resultou em sua estigmatização sociocultural^(32)^. Essa percepção social distorcida contribui para a desvalorização na escolha do curso, por vezes considerado de menor prestígio, em desacordo com a real complexidade e relevância de suas contribuições à saúde integral dos indivíduos^(33)^.

Além das questões mencionadas, os estudantes desta área enfrentam obstáculos de ordem estrutural, especialmente relacionados à organização curricular e à realidade socioeconômica. A grade curricular (de caráter integral no local deste estudo) e a dificuldade de conciliar os estudos com trabalho remunerado, somadas à escassez de auxílios financeiros, impõem barreiras adicionais, sobretudo para estudantes em situação de vulnerabilidade socioeconômica, que dependem quase exclusivamente de redes de apoio para garantir a permanência no curso^(34)^.

Complementarmente ao currículo obrigatório, o envolvimento em atividades extracurriculares (com pouca participação neste estudo), apesar de ser uma cobrança adicional, contribui para o fortalecimento do senso de pertencimento e identificação com a profissão, ao favorecer o desenvolvimento da autoconfiança nas competências técnicas e teóricas fundamentais para a inserção no mercado de trabalho^(35)^.

Quanto aos fatores protetivos, os estudantes pontuaram uma boa rotina de exercícios físicos e dedicação ao lazer, mas, ainda assim, foram observados elevados níveis de estresse. Tais resultados contrastam com evidências presentes na literatura, que indicam essas práticas como atenuantes ao sofrimento psíquico. Contudo, variáveis como o tipo de atividade realizada, a quantidade de tempo dedicado e a forma de engajamento podem influenciar a percepção de relaxamento^(36-38)^.

Aliado a esse cenário, a qualidade do sono foi considerada insatisfatória pelos participantes deste estudo. Nesse sentido, evidências científicas apontam que o sono insatisfatório pode comprometer significativamente o desempenho acadêmico, além de exercer efeitos adversos sobre a saúde mental. Tais impactos incluem níveis elevados de estresse, prejuízo na concentração e redução do otimismo, que influenciam o rendimento e a percepção sobre o curso escolhido^(39,40)^.

Entende-se ainda que a qualidade do sono é impactada negativamente pela exposição contínua a estímulos digitais decorrente do uso massivo de telas para fins acadêmicos e sociais, o que predispõe a respostas disfuncionais ao estresse e compromete o estado emocional^(41)^. Ademais, as redes sociais, em particular, contribuem para o aumento de sintomas ansiosos, pois funcionam como formas de escapismo. Ao retornar à realidade, a pessoa sente-se improdutiva, com gestão de tempo ineficiente e angustiada^(42)^.

Embora a literatura não estabeleça uma idade específica para a análise e modulação das estratégias de enfrentamento, sugere-se que a exposição precoce ao tema pode contribuir para a consolidação de padrões comportamentais mais adaptativos para o autoconhecimento e desenvolvimento pessoal^(3)^.

Diante desta análise e discussão, reforça-se a relevância de instituições públicas de ensino superior, mais especificamente dos Departamentos de Enfermagem, adotarem um plano de cuidado integral à saúde dos discentes, com vistas à ampliação da oferta de suporte psicopedagógico e psicológico voltado à promoção de estratégias de *coping* mais eficazes e ao fortalecimento das competências socioemocionais.

Adicionalmente, é crucial capacitar os docentes para que reconheçam e acolham as vulnerabilidades entre os estudantes, garantindo a detecção precoce e o encaminhamento eficaz deles para os serviços de apoio, bem como a inserção de atividades voltadas à promoção do bem-estar na grade curricular. Assim, ao favorecer o desenvolvimento de recursos internos de enfrentamento e suporte estrutural, essas iniciativas podem contribuir para a formação de profissionais mais autônomos e mentalmente saudáveis, em consonância com a quarta meta do terceiro ODS^(10)^.

### Limitações do estudo

Este estudo apresenta algumas limitações que devem ser consideradas na interpretação dos resultados. A amostra foi composta por um número reduzido de estudantes de uma única instituição pública, o que pode restringir a generalização dos achados para outras realidades acadêmicas. O desenho transversal permite apenas a identificação de associações, e não estabelece relações causais entre as variáveis avaliadas. A coleta de dados, por meio de questionários autorrelatados, também pode estar sujeita a vieses, como a interpretação dos questionários. Além disso, o contexto específico pós-pandêmico, associado à greve docente, pode ter influenciado os níveis dos sintomas de depressão, ansiedade e estresse, dificultando a separação dos efeitos dessas condições em relação a fatores habituais do ambiente acadêmico.

Para pesquisas futuras, sugere-se a realização de estudos longitudinais que avaliem a eficácia de intervenções para a promoção de *coping* funcional, monitorando suas repercussões nos sintomas de depressão, ansiedade e estresse em uma amostra maior.

### Contribuições para a área da saúde, enfermagem ou políticas públicas

Ao identificar níveis significativos de sintomas depressivos, ansiosos e de estresse, e o uso de estratégias de *coping* com foco emocional entre os estudantes de enfermagem de universidade do estado do Maranhão, Brasil, associados à análise dos dados sociodemográficos, o estudo oferece subsídios importantes para a elaboração de políticas institucionais que promovam o suporte à saúde integral dos estudantes, com foco na saúde mental. Assim, esse diagnóstico situacional pode incentivar a busca por estratégias de intervenção direcionadas à mitigação do quadro de sofrimento psíquico entre os estudantes e contribuir indiretamente para o aprimoramento dos programas de formação em enfermagem, que devem prezar pelo bem-estar integral dos futuros profissionais, para que estes enfrentem com mais autonomia as adversidades durante as vivências universitárias e estejam mais adaptados às demandas provenientes da atividade profissional.

## CONCLUSÕES

Este estudo demonstrou a presença significativa de sintomas de depressão, ansiedade e estresse entre estudantes de enfermagem, ao passo que destacou a predominância de estratégias de enfrentamento disfuncionais. Desse modo, enfatiza-se a necessidade de ações institucionais que promovam o desenvolvimento de habilidades psicossociais e de inteligência emocional, a partir da implementação de programas de apoio psicopedagógico e psicológico efetivos, contínuos e acessíveis, bem como atividades que valorizem a identidade profissional e promovam o bem-estar, visando à formação de profissionais de enfermagem com maior domínio psicoemocional e ao alcance da quarta meta do terceiro ODS.

## Data Availability

Os dados de pesquisa estão disponíveis no corpo do artigo.
